# Development and evaluation of an extra-curricular programme focussing on high impact career opportunities for medical professionals

**DOI:** 10.1371/journal.pone.0284856

**Published:** 2023-04-24

**Authors:** Akhil Bansal, Joseph Pusey, Rahul Shah, Abraham Tolley

**Affiliations:** 1 Faculty of Medicine and Health, University of Sydney, Sydney, New South Wales, Australia; 2 St Vincent’s Hospital, Darlinghurst, New South Wales, Australia; 3 Bolton NHS Foundation Trust, Bolton, United Kingdom; 4 School of Clinical Medicine, University of Cambridge, Cambridge, United Kingdom; Tallinn University: Tallinna Ulikool, ESTONIA

## Abstract

**Background:**

Many medical professionals seek to do good through their careers, which may involve pursuing non-clinical options such as research, policy, or education in addition to clinical work. Working out which paths will lead to the largest social impact is a challenging question and of interest to many doctors. However, there are few, if any, services that use an impact-oriented framework to support doctors who want to make career decisions based on impact.

**Objectives:**

To describe the development of an 8-week fellowship programme to introduce medical professionals to careers paths and focus areas which could lead to a particularly large social impact. And to evaluate the programme in terms of engagement, utility, changes in knowledge and career attitudes of participants.

**Methods:**

The ADDIE instructional design model was used to design and evaluate this fellowship programme. An 8-week curriculum was designed by medical professionals and delivered to medical students and doctors around the world utilising a flipped learning style. Quantitative and qualitative data on the programme were collected and analysed.

**Results:**

There was more demand for the programme than anticipated. We found that the fellowship was engaging and useful to medical students and doctors. It resulted in an increase in knowledge and skills on how to consider impact in one’s own career and a change in participants’ attitudes and behaviours, with some participants making changes to their career and charitable giving following the programme.

**Conclusions:**

We believe an impact-orientated, practical co-curricular programme is valuable to medical professionals exploring impactful career options and there is demand for further programmes in this space.

## Introduction

The desire to help others and to have social impact is one of the most common motivations expressed by those seeking to become doctors [1–4]. In the public imagination, and that of most doctors, this desire is likely to be fulfilled through a career of clinical practice- working in hospitals and community settings to diagnose and treat patients. However, many doctors choose to spend a proportion of their time working in a non-clinical capacity [5]. This work can include clinical research, education or management tracks to doctors choosing to leave medicine entirely. The reasons for these decisions vary, and include personal circumstances, alternative career ambitions, and increasingly, exhaustion and burnout [6–9].

Doctors may also explore non-clinical work as they are seeking to increase their social impact. A medical background lends itself to many aligned fields which have the potential to help others, for example medical research, policy or education. Some of the interventions that have resulted in the huge gains in in patient outcomes in recent times have arisen from doctors working in non-clinical fields of policy or research, such as the Surgical Checklist pioneered by Dr Atul Gawande [10–12] or RNA vaccine technology led by Drs Sahin and Tureci [13]. Attempting to compare the impact of different medical career paths is challenging and a relatively uncommon idea–some analyses have suggested that there may be the potential to save more lives than an average doctor practicing in an economically developed country through practising in low and middle income countries [14], making substantial donations to charities, or working in areas such as research [15–17].

There are currently few (if any) services dedicated to supporting doctors who are keen to explore career paths with consideration to social impact, in areas such as education, policy, entrepreneurship and research.

To meet this need, we developed an 8-week fellowship programme designed specifically to introduce medical professionals to career paths and focus areas could lead to a particularly large social impact. This programme utilised prioritisation and impact framework inspired by the effective altruist philosophy [18] and used by organisations such as grant makers Open Philanthropy [19] and charity evaluator Givewell [20] and brought together academic reading and testimonies from medical professionals. Areas explored included medical research, global health, health policy, biosecurity, medical technology and charitable giving. The use of co-curricular programmes to increase work-related skills and knowledge and stimulate personal professional development is well established [21, 22]; however, to our knowledge, there are no examples in the literature of such a programme being used for this particular purpose.

The aim of this study was to evaluate a fellowship programme that aims to introduce medical professionals to impactful career frameworks and opportunities, and assess its impact in terms of changes of engagement, utility, changes in knowledge and career attitudes of participants. This article describes the design and implementation of our novel fellowship programme, focussing on an impact-oriented framework for medical careers, and evaluates the impact of the programme in line with the key aims. We found that the fellowship programme was engaging and effective in encouraging medical professionals to adopt an impact-focussed approach to career opportunities.

## Methods

The fellowship followed the ADDIE (Analysis, Design, Development, Implementation, Evaluation) instructional design model [23] for its design and evaluation.

### Analysis

In the analysis phase, a brief literature review was conducted to understand what similar programmes already exist. A “fellowship”, referring to a learning programme involving regular facilitated peer-to-peer themed discussions inspired by curated reading material which we evaluate here, has not been previously discussed in the literature to our knowledge. However, medical ‘book clubs’ offer a close proxy, and we wished to emulate aspects of their design such as their relative informality, well-trained and effective facilitators, and practical application to professional development. These were shown to foster engagement with the subject material and forge professional connections [24].

As the fellowship aimed to inspire medical students and doctors to consider impact-based aspects of their future careers, the literature on career planning interventions for early-stage clinicians was also considered. The vast majority of such interventions were related to very conventional medical careers and were specialty-specific [25–27]. Our programme sought to be broader in scope and less didactic than these reviewed interventions, we employed certain aspects of these programmes. For example, we provided opportunities for mentoring and networking similar to these interventions, since mentored medical students are shown to be more research-productive and perform better in medical school, along with demonstrated higher well-being [28].

To determine the topic areas this fellowship focussed on, a prioritisation and impact framework inspired by the effective altruist philosophy [18] and used by organisations such as grant makers Open Philanthropy [19] and charity evaluator Givewell [20] was employed. The topics of medical research, global health, health policy, biosecurity, medical technology and charitable giving were identified as particularly impactful and neglected areas that were worth focussing on.

To inform the content of the fellowship, we reviewed the academic literature discussing each of these cause areas and many of the relevant articles featured in the core curriculum content for each week. It was clear that although unifying educational programmes like ours that considered impactful cause areas such as health policy, global health, biosecurity and medtech have not been run in the past, the necessity of educational programmes in each of these separate fields have been reviewed [29–34]. All of these studies conclude with a plea to plug knowledge gaps in traditional medical school curricula in these fields, and encourage interest in the cause area due to the scale of the problem: this added to the impetus in designing our fellowship.

In addition, a variety of medical students (n = 10) and doctors (n = 7) were interviewed to understand prospective learners’ current knowledge and skills, as well as their needs. Further, interviews were conducted with experts (n = 4)—individuals who had previously designed similar fellowship programmes—to inform programme design. Together, the literature review and scoping interviews were used to inform the aims and design of the course, as well as the included content.

The key areas and gaps identified through this analysis, which informed subsequent design were:

1. Uncertainty about which fields presented greater than average opportunities for impact: Although individuals knew that the impact of work in different areas varied, they were unsure where they should work if they wanted to have a particularly large positive impact.
2. Poor knowledge of opportunities available to doctors beyond direct clinical work: Most individuals were unaware of feasible options available for doctors to work in fields such as research, policy and entrepreneurship, either alongside or instead of clinical work.
3. How to balance moral and personal considerations with career planning: those interviewed frequently mentioned stress that arose when balancing personal factors such as location or income, as well as moral factors such as volunteer tourism and concerns about negatively affecting the healthcare system, with seeking to increase the impact in their careers.

Based on this, the key objectives of the fellowship programme were to:

1. Provide information about the impact of different opportunities that medical professionals can pursue, and assess their impact
2. Provide information about the opportunities available to medical students and doctors to work in these areas and, with examples, assess why doctors may be able to contribute within them meaningfully
3. Support and encourage participants to make holistic career decisions that consider impact as a key factor, including in their charitable donations

### Curriculum design

Based on the analysis above, and given that medical students were geographically spread out and preferred flexibility of timing, it was determined that a flipped learning style [35] was most appropriate for the programme structure. “Flipped classroom” teaching involves students familiarising themselves with content through curated reading and interactive videos, before attending focussed teaching sessions and has been demonstrated to be s superior to traditional formats in terms of student learning [36, 37]. Given that students and doctors wanted a programme which would allow them to discuss ‘key gap 3’, we decided this format would be most conducive to facilitated informed discussions for learning objectives 2 and 3, rather than a more traditional didactic approach. The ways to enhance the effectiveness of flipped classroom approaches have recently been reviewed [38]. These reviews recommended interventions such as structured learning processes, prolonged duration of flipped classroom, in- and out-of class problem solving activities, peer work, weekly review of key concepts and facilitator training, which were subsequently employed in the implementation phase of the fellowship.

Based on feedback of likely time availability and level of interest of potential participants, key readings and resources of approximately 2–4 hours in length were provided to participants in advance of weekly 1.5-2-hour discussion groups for further conversation. The fellowship was 8 weeks in total. The pre-reading was designed to appeal to medical students and junior doctors with an interest in increasing the impact of their medical careers. The discussion sessions took place in person or virtually, in small groups of 4–8 participants with 1 instructor (facilitator).

The themes of each week, and the learning objectives, are presented below in Table 1, and are modelled based on areas identified in the analysis phase with reference to the overall key objectives of the fellowship programme.

**Table 1:** Themes and summarised learning objectives of each week of the fellowship.

Week	Title	Learning outcomes
1	Doing good, clinical medicine and considering your impact	1. Consider what it means to do good and different philosophical frameworks for considering ‘good’ and ‘impact’ 2. Introduce the importance-tractability-neglect framework and discuss what some of its strengths and limitations are 3. Consider medical research as a way of addressing some of the world’s most pressing problems
2	Global Health	1. Explore global health and development and some of the most important areas within it 2. Evaluate the suitability of global health as a cause area for doctors to work on 3. Explore how to contribute to global health as doctors and medical students
3	Health policy	1. Introduce health policy, discuss its importance and some of its limitations 2. Discuss different roles medics can play in health policy 3. Explore the concepts career capital and credibility and how they lend credence to doctors being involved in health policy advocacy and activism 4. Explore how to contribute health policy with examples
4	Biosecurity	1. Introduce the risks posed by novel emerging pathogens in the past and future 2. Explore the threat biotechnology may pose e.g. through bioweapons or research accidents 3. Explore work mitigating these threats on levels of molecular research and public health 4. Consider health security and biosecurity in the context of global catastrophic risks 5. Explore how to contribute to biosecurity as doctors and medical students
5	Technology in Medicine	1. Introduction to artificial intelligence and machine learning and the current status of medical artificial intelligence 2. Discuss some particular promising areas of technology in medicine, including: applications of medical technology in low resource settings, telemedicine 3D printing 3. Explore some of the major roadblocks to adoption of novel medical technologies, and some of the reasons you may or may not want to get more involved in this cause area. 4. Explore how to contribute to technology in medicine as doctors and medical students
6	Mental health, wellbeing and chronic pain	1. Introduce mental health, wellbeing, and chronic pain and explore why they might be high impact cause areas 2. Explore the global burden of mental health problems and of chronic pain, metrics for subjective wellbeing and happiness as well as interventions in both areas and their potential effectiveness 3. Consider how to go about identifying potential cause areas in general, and how to find which interventions are the highest priority in each
7	Effective giving and earning to give	1. Discuss the idea that charities can differ significantly in their cost-effectiveness, and introduce charities that may have higher impact 2. Consider our personal cause prioritisation in how we choose to spend our money and how/ why that may differ from our cause prioritisation in how we spend our time 3. Consider the idea of giving effectively—what is it, how much could we give and where; how one can ‘earn to give’ and whether one should 4. Introduce the concept of value drift and discuss giving pledges [39]
8	Capstones and next steps	1. Write a short report (capstone project) on something that the fellowship has made you excited to explore more or think about in your career and present it to the other fellows in your group 2. Reflect on the fellowship; medicine; personal fit for different career opportunities & next steps

### Curriculum development

After the curriculum objectives had been determined, the content of each week was created through a collaborative, co-design format, whereby the reading materials were compiled by medical professionals working in relevant fields to the week’s objectives. The choice of materials was based on literature reviewed and the needs identified in the analysis phase. Reading materials were taken primarily from academic journal articles, published books and blog posts. Materials were then reviewed by facilitators, experts and potential prospective participants. The course material was developed on word processor, with additional audio-visual material recorded in the form of podcast interviews and integrated into the written material.

The instructional materials were not pre-tested although all materials were reviewed by facilitators, experts and potential prospective participants. This is because this programme was itself the pilot programme, designed to test the and the fellowship programme, on a small cohort.

### Curriculum implementation

After the curriculum was created, the fellowship was implemented for the first time over 8 weeks in February-April 2022. The fellowship was run through High Impact Medicine [40]) an organisation and network of medical students and professionals thinking about how to do the most good with their careers.

#### Fellowship participants

Doctors at any stage of medical training and medical students in all years of their degree were eligible to take part in the fellowship, Recruitment was conducted through High Impact Medicine’s existing networks of medics as well as through publicity channels of medical schools within the UK and Germany. The fellowship had a competitive application process, which included both closed and open-ended questions; inclusion criteria for selection were alignment with fellowship goals, strong academic track record, ability to engage with example subject material intelligently and time availability to take part in the fellowship. Exclusion criteria were insufficient understanding of the purpose of the fellowship or lack of time commitment to take part in the fellowship. Two independent course facilitators reviewed each application, with an aim of inviting approximately 20–30 participants into the programme.

#### Facilitators

Facilitators were invited by application and chosen by the programme coordinators. They were selected based on their knowledge of high impact opportunities within and outside clinical medicine, time availability and prior medical teaching experience.

Facilitators were trained by the course designers in a 2-hour session and were supported throughout by course organisers. Facilitators met twice virtually during the fellowship to discuss lessons learnt and feedback to improve the remainder of the fellowship.

#### Fellowship mode of delivery

Each week, participants met in small groups of 4–8 fellows and a facilitator. A workbook was distributed to participants before the beginning of the programme. The workbook consisted of approximately two to three hours of pre-work for each week, consisting of readings related to the week’s objectives (Table 1) as well as short activities for participants to complete.

### Evaluation

The course was evaluated by facilitators and fellows in both a formative and summative fashion. The purpose of the formative evaluation was to assess fellows engagement with the programme, and for quality improvement. The summative evaluation was used to inform the results of this study, including the fellowship and impact evaluations.

#### Formative evaluation

Formative feedback involves improving each part of the ADDIE process. The following formative feedback steps informed the evaluation of the fellowship

1. The learning objectives in fellowship design were improved following feedback from potential participants and facilitators.
2. The readings and tasks for each week as part of the workbook went through rounds of feedback from both experts in the area, potential participants and facilitators.
3. In each week of the fellowship, there were activities where participants could share feedback on topics, which were noted by facilitators and shared with other facilitators. Facilitators also kept in contact during the fellowship using Slack to discuss feedback and lessons learnt. For example, particularly useful exercises used by one facilitator were often used by other facilitators subsequently.
4. In the final week of the fellowship, fellows were invited to present on a topic that was of interest to them. This allowed facilitators to see the depth of their fellows’ engagement and assess their preferred topics informally.

#### Summative evaluation

Quantitative and qualitative data were collected from participants through a post-programme questionnaire. As there was no available, validated instrument for assessing such co-curricular programmes, we generated our own questionnaire, based on the aims of the evaluation identified above.

The form contained 24 questions, including both closed and open-ended questions, categorised into 8 domains which each related to three of the four levels of Kirkpatrick’s evaluation model—evaluation of fellows’ satisfaction to the course, evaluation of how much fellows learnt over the fellowship and evaluation of how much their future behaviour changes following the fellowship. The full questionnaire can be found in S1 Table.

The post-course questionnaire was delivered online via Qualtrics Survey. Fellows consented through a virtual consent form before they were able to complete the questionnaire.

Fellows were given time to complete this survey in the final fellowship session, and subsequent reminders were sent out to each group to encourage responses, in order to maximise response rates. As the survey was anonymised, participants who did not complete it were unable to be followed-up individually.

### Data analysis

Quantitative data were analysed using descriptive statistics on Google Sheets [41].

Qualitative data were analysed with content thematic analysis: the key themes were identified from all answers to open ended questions, and the most frequently mentioned ideas included in results below. Furthermore, particularly useful quotes were included in results tables.

A full anonymised dataset of quantitative and qualitative survey responses can be found in S1 Table.

### Ethics approval

The University of Sydney Human Research Ethics Committee approved the study (2022/766). Written consent for participation was obtained from participants to enable us to include their data from this study.

## Results

After a competitive application process that saw 72 people apply to the fellowship, a total of 55 individuals were accepted and 52 participated in the full programme. Of those, 42 participants completed the post-fellowship questionnaire (response rate 80.7%). Participants were a mixture of pre-clinical medical students, clinical medical students, and junior doctors (Table 2); most were based within the UK and Germany, although participants were located in 7 different countries overall.

**Table 2:** Demographic characteristics of fellowship participants (n = 42).

	Number (n, (%))
**Country of location**	
United Kingdom	27 (64.3%)
Germany	7 (16.7%)
Australia	3 (7.1%)
Iran	1 (2.4%)
Singapore	1 (2.4%)
Sweden	2 (4.8%)
Switzerland	1 (2.4%)
**Gender**	
Male	24 (57.1%)
Female	18 (42.9%)
Non-binary	0
Prefer not to answer	0
**Stage of medical training**	
Early-stage medical student	17 (40.5%)
Late-stage medical student	15 (35.7%)
Junior doctor	8. (19.0%)
Mid-career doctor	2 (4.8%)

Early-stage medical student referred to people in the first half of medical degree, late-stage referred to studies in the second half of their medical degree, junior doctors were participants in year 0–3 post graduation, and mid-career doctors were more than 3 years post-graduation

### Response to and engagement with programme

**Fig 1:**
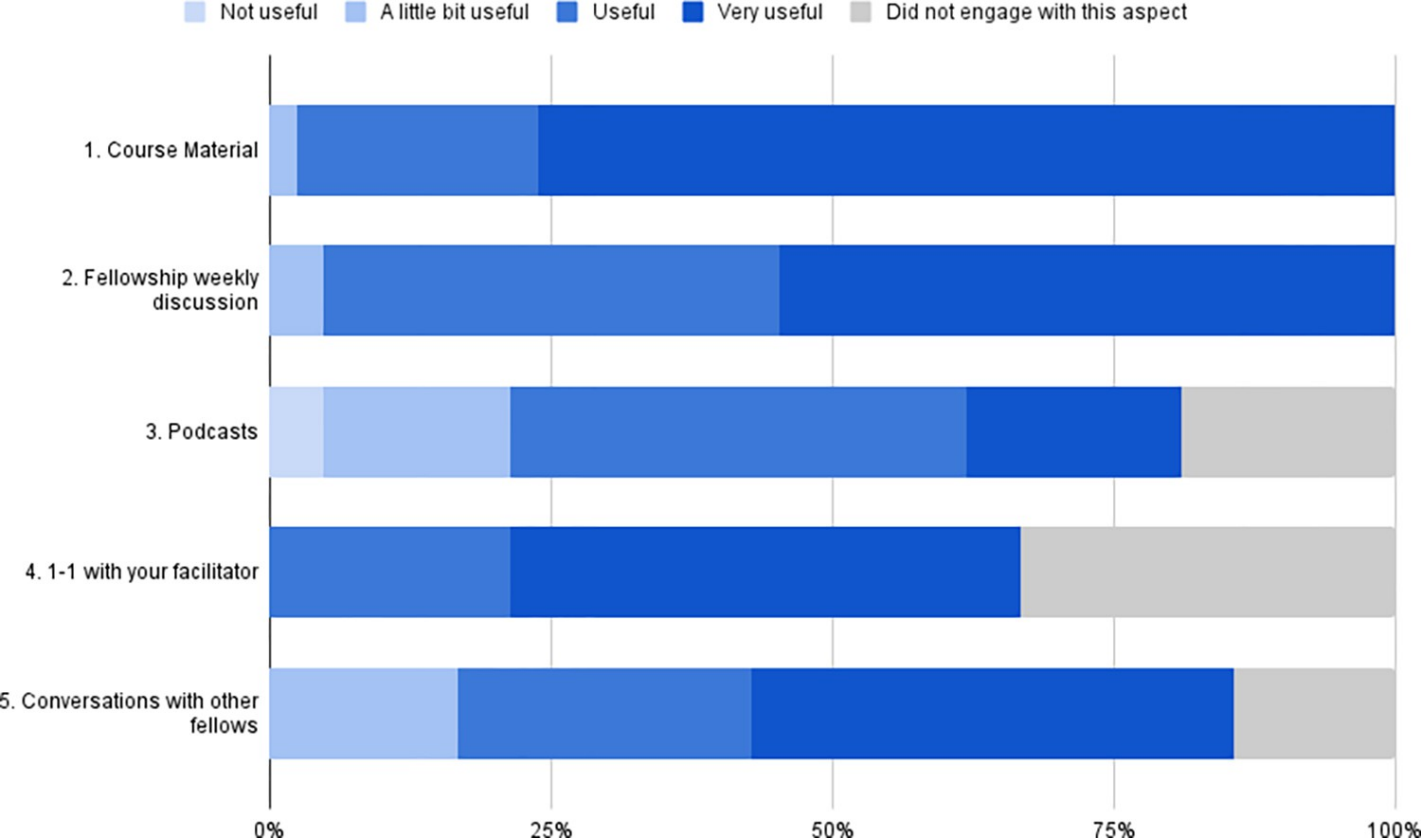
Participant engagement with and perceived usefulness of different aspects of the fellowship.

Overall, participants found the programme useful (Table 3) and engaged with most aspects of the programme (Fig 1). The course material and weekly fellowship discussion were well-received by fellows, with 97.6% (n = 41/2) and 95.2% (n = 40/42) finding them useful or very useful respectively. Of those participants who did have 1–1 conversations with their facilitators (n = 28/42), all found them useful or very useful. Participants found all the aspects of the programme to be at least a little bit useful, other than a small number of participants found the podcast (n = 2) to not be useful. In the open-ended questions about which aspects of the programme being found the most useful (Table 3), the course material and weekly fellowship discussions emerged as frequent themes; as well as this, participants valued the connection to like-minded individuals, and the breath of the topics covered through the fellowship.

**Table 3:** Areas of the fellowship that the participants found most useful.

Theme	Example quotes
Course material was informative and covered a broad range of topics	"Extremely well-researched reading that was very broad" "The most useful part was probably the reading that we did each week because it really broadened my knowledge base "
Weekly discussions with a group offered access to a variety of different experiences and expertise	“The weekly discussions were a lot fun. Especially talking about different aspects everyone came into contact with, because this differed widely. Additionally, bringing in a broad variety of experience and expertise from different sections was very helpful!?” “I liked the sessions. It was more like flipped classrooms, and more student-centered than lecture. Also, it gave the participants the chance to challenge their ideas.” “The group sessions was the best way to communicate and debate about different aspects of every subjects”
Connection to a community of people with similar thoughts and valued	“I valued connection to likeminded doctors worldwide…” “The connection to like minded people who were thinking similar thoughts was the best part of the programme” “[I valued] connecting to a network of medical students at my university who are interested in similar things to me”

The participants also suggested several areas for improvement (Table 4), which fell into two main themes: more advice on opportunities after the fellowship as well as more in person socials during and following the fellowship.

**Table 4:** Areas of improvement for the fellowship.

Theme	Example quotes
Participants wanted more advice on post-fellowship opportunities and advice on how to pivot their careers	“Have a concrete way for us to engage with Hi-med rather than simply discussion” “I think maybe forming some steps to take forward (e.g. blog post/teaching session/some form of advocacy/education/action) might be quite helpful?” “I would be pleased to get engaged in some research project or career advise, to be honest, I don’t have a certain plan right at the moment but am highly motivated to search for future perspectives!” “I think finding a concrete way of us being involved in research/other projects will be beneficial (preferably working together with other peers)”
Participants wanted more social and in-person events	“A few more in-person events / a mix of online and in-person activities.” “Obviously understandable that we couldn’t do much in person but in person discussions as well as some interaction with other groups”

### Knowledge and skill gained

The fellowship also had some effect for all participants on their knowledge about impactful opportunities within medicine (Table 5)- for most, this effect was either moderate (n = 17/42, 40.5%) or major (n = 19/42, 45.2%). There was a reasonably even distribution between different areas that the participants felt that they learnt the most about, with medical technology and AI (n = 29, 69.0%) and biosecurity (n = 23, 54.8%) the most frequently given answers. In their open-ended responses (Table 6), participants noted that they learnt about new career options that they were previously unaware of, that the programme taught them about how and motivated to align their career with impact, and that they found some specific information about certain cause areas and organisations helpful.

**Table 5:** Impact of fellowship on new information and knowledge gained.

Question	Number (n, %))
**What areas did you learn the most new information about?**	
Global health	17 (40.5%)
Health policy	20 (47.6%)
Biosecurity	23 (54.8%)
Medical technology and AI	29 (69.0%)
Mental health and wellbeing, chronic pain	13 (31.0%)
Effective giving and earning to give	19 (45.2%)
**How much of an effect did the fellowship have on your knowledge about high impact opportunities within medicine?**	
No effect	0
Small effect	6 (14.3%)
Moderate effect	17 (40.5%)
Major effect	19 (45.2%)

**Table 6:** Participant responses about their main learnings from the fellowship.

Theme	Example quotes
New possible high impact career options for doctors	“There are many more opportunities out there that are high impact with some that I had never really thought about before” “This has really given me some clarity and ease of mind in thinking about future career choices. It has given more than a clear framework; it has given a good and rounded introduction to a whole host of topic areas. The balance of giving moral/ethical guidance with giving tangible information has really settled the anxieties that can come with career planning!” “There are many more opportunities out there that are high impact with some that I had never really thought about before”
Aligning career more closely with impact	“I feel obliged to triage my own career choices by possible impact” “How to find empirically high impact areas.” “I reconsidered the impact I might have as a doctor and am looking at how to
Learning about specific areas or organisations of interest	“I am considering applying for some of the policy opportunities presented in the fellowship” “Learnt more about mental health and chronic pain” “I learnt more and want to apply for the Charity Entrepreneurship programme”

### Change in career behaviour and decisions

We assessed the anticipated changes in career behaviour and decisions that our fellowship had on fellows (Table 7). A majority said that it had a moderate or major effect (n = 36, 85.7%) on the way they think about their future career, and over half (n = 22, 52.4%) said that it had a moderate or major effect on the way they will think about their future donations. Participants said the programme has widened the options that they would consider in their careers, and made a few participants change the career direction to focus on areas that they perceive to be impactful (Table 8). 2 participants took a Giving What We Can(39) pledge to give 10% of all their future income to highly effective charities, and a further 2 participants took a trial pledge to give 1% of all their future income to highly effective charities. Several (n = 6) members also stated that they planned to take the Giving What We Can pledge.

**Table 7:** Impact of fellowship on how participants perceive their careers.

Question	Number (n, (%))
**What effect did the fellowship programme have on the way you think about your future career?**	
No effect	1 (2.4%)
Small effect	5 (11.9%)
Moderate effect	24 (57.1%)
Major effect	12 (28.6%)
**What effect has the fellowship had on the way that you think about your future donations?**	
No effect	9 (21.4%)
Small effect	10 (23.8%)
Moderate effect	12 (28.6%)
Major effect	10 (23.8%)

**Table 8:** Participant responses about how the fellowship changed their perception of their careers.

Theme	Example quotes
Changed the career direction of several participants to areas that they perceive to be impactful	“I have decided to focus my research on pandemic preparedness and biosecurity, and will enter this area after graduation” “I decided to shift towards the cause area of animal suffering” “I am now doing more work in health policy”
Widened the career options that participants are considering	“It has made me re-think the ways that I can change my career to work on something where I may have a higher impact on things.” “This fellowship has really made me consider about working abroad, in a Doctors without Borders sense, but not just that as a global doctor making connections with other doctors around the world so we can all learn from each other and elevate global health and improve lives.”

## Discussion

This study evaluated a novel and newly designed fellowship programme which introduced medical students and professionals to an impact-oriented framework through which they could consider their career and its impact. We found the programme to be engaging and useful to medical students and doctors, which successfully encouraged most participants to change the way they thought about their careers and charitable giving.

### The benefits of an impact-oriented fellowship programme for medical professionals

The benefits of our fellowship programme were validated throughout the design, implementation, and evaluation of this fellowship programme. A needs analysis showed that both medical students and doctors were interested in exploring impact-oriented approaches to careers, but there are very few websites and programmes that helped medics explore different pathways and fewer still that had an explicit rationale behind the recommendations they made. In implementing the programme, we found significantly more people interested than we had anticipated leading us to expand the number of cohorts to accommodate over 50 fellows (we had originally planned for 20–30 when developing the programme).

In the evaluation of the programme, we found that the fellowship programme was well received and that it achieved its desired outcomes- On Kirkpatrick’s hierarchy of evidence, we found that the programme was largely well-received (Level 1), resulted in appreciable increased in knowledge and skills about how to consider impact in one’s career (Level 2), and that the programme did lead to changes in attitudes and behaviours of participants towards their career (Level 3).

Our study provides evidence that further iterations of this fellowship or similar programmes may provide further, similar benefits to medical students and doctors. Such programmes may help doctors who are looking to do some work outside of clinical practice, by circumstance or choice, make informed decisions to increase the impact of their work and feel supported in pursuing such opportunities.

There is a growing body of work demonstrating that the NHS is facing a trend of junior doctors leaving the organisation before their specialty training is complete: in 2011 the proportion of F2 (Foundation Year 2) doctors progressing into specialty training was 71.3%, falling to 37.7% in 2019. Burnout is a very commonly cited reason for leaving UK medicine and has been identified as a theme in semi-structured interviews conducted amongst doctors that have left the NHS [42, 43]. Ensuring work is engaging and feels meaningful has been shown to empower doctors and reduce burnout–the content of impact-oriented programmes such as this one can help facilitate this process. Furthermore, encouraging physician group study with discussion groups, such as took place during the fellowship, is a recognised prevention technique [44] to manage burnout. Finally, not having adequate broad academic and clinical learning opportunities and mentorship during the Foundation Training programme are also commonly cited reasons for leaving the NHS [42]; by encouraging an exploration of sub-fields within medicine such as research, education and management during the fellowship, as well as providing opportunities to connect with individuals who have pursued such paths, this programme can help fill an area with unmet need.

### Value of co-curricular programmes for medical professionals

In designing the fellowship, our analysis showed that an action-oriented programme was the most likely to engage medical students and doctors, a busy professional group. Therefore, whilst the fellowship programme’s pre-reading material featured some theoretical foundational reading, it aimed to be as pragmatic as possible. Specifically, the pre-reading material and discussion focussed on helping participants assess their personal fit for particular types of work and cause areas, including relevant organisations working in relevant fields.

The format of our fellowship resembles book clubs, similar co-curricular program designed to develop skills and knowledge amongst a professional group and have been used for professional development extensively. Even amongst busy doctors, evidence suggests that formats such as book clubs can facilitate reflection, bonding with peers and development of community, and engagement with the reading material [45, 46]; our findings add to this body of evidence.

Other features of the fellowship that were well received and may provide value to busy working professionals were: one-to-one calls where we were able to discuss participants’ future plans in greater personal depth, and the podcasts from medical professionals working in the areas highlighted during the programme, exploring how they had gone about making career decisions. The benefits of multimedia learning, as was adopted by the fellowship, to accommodate for varied learnings styles and to improve engagement is well-known [47], as is the value of peer-to-peer learning, to improve increase confidence in understanding the subject material and facilitating community connections [48].

Finally, some participants took significant steps as a result of their fellowship, such as stating intentions to re-orient the focus of their career or, more tangibly, taking a Giving What We Can [39] giving pledge. Although we cannot claim sole causality, as such participants may have been considering such actions prior to the fellowship, it seems likely the fellowship’s content contributed to their decision making. Participants also highlighted that they would like to see further opportunities to take the ideas discussed in the fellowship forward, demonstrating demand for further action-oriented programmes.

We believe that action-oriented, practical co-curricular programmes featuring a variety of forms of content including one-to-one conversations may be particularly useful and well-received by medical professionals exploring impactful career options.

### Limitations and future directions

One limitation of our study is that our evaluation was undertaken immediately after the programme, and it is therefore difficult to ascertain the long-term impacts of the programme. A further limitation was that there was no information on the perspectives of participants who did not complete the survey, who may represent a cohort who were less satisfied by the programme. Finally, we did not conduct a survey of participants prior to beginning the programme, which would have allowed shifts in perspective to be more accurately seen compared to purely post-hoc reflections.

Looking forward, we would like to undertake a follow-up study of fellowship participants 6–12 months after taking part in the programme, to understand whether and how the effects of the programme persisted, and whether the intent to make career changes translated into action. In conjunction to this, we would like to improve the content of the fellowship in response to feedback, for example adding additional support and opportunities on pursuing different impactful projects into the existing programme or developing more in-depth programmes, in response to unmet participant need. We would also be interested in exploring whether this fellowship programme is scalable, when delivered to larger number of participants, and how it may need to be adapted for participants in countries with different health systems and socioeconomic contexts. Future work could explore whether similar programmes may be needed by and beneficial to other professional groups and whether a similar action-oriented framework would be particularly valuable.

## Conclusion

We have designed and, in this study, evaluated a novel fellowship programme that introduced medical professionals to impact-focused framework and explored career options that may have a large, positive social impact. We found a significant number of medical professionals were interested in such a programme, and that it was well-received. Future programmes should further consider how to help participants translate the knowledge and skills gained through such programmes into tangible actions and career changes.

## Supporting information

S1 Table Full anonymised dataset. Spreadsheet contains qualitative and quantitative survey responses.
(XLSX)
